# Prognostic factors in intrauterine insemination cycles

**DOI:** 10.5935/1518-0557.20180002

**Published:** 2018

**Authors:** Fernanda Sicchieri, Aline Bomfim Silva, Ana Carolina Japur de Sá Rosa e Silva, Paula Andrea de Albuquerque Sales Navarro, Rui Alberto Ferriani, Rosana Maria dos Reis

**Affiliations:** 1 Sector of Human Reproduction, Department of Gynecology and Obstetrics - Ribeirão Preto Medical School, São Paulo University, Brazil.

**Keywords:** intrauterine insemination, sperm motility, pregnancy rate

## Abstract

**Objective:**

This study aimed to evaluate the clinical pregnancy rate of intrauterine insemination cycles in relation to patient age, cause of infertility, ovulation induction method, number of mature follicles and sperm with progressive motility.

**Methods:**

This retrospective observational study included 237 intrauterine insemination cycles performed from 2011 to 2015 at the Assisted Reproduction Service of the Hospital das Clínicas of the Ribeirão Preto Medical School, University of São Paulo. Student's t-test was used to compare quantitative variables and the chi-square test was used to compare qualitative variables.

**Results:**

Patient age was inversely and significantly correlated with pregnancy rates (*p*=0.001) (Pregnant women = 32.56±5.64 years, non-pregnant women = 36.64±5.03 years). Cause of infertility, ovulation induction method, number of mature follicles and sperm with progressive motility were not associated with pregnancy rates. The overall clinical pregnancy rate was 7.59%. In the subgroup of patients (n=102 cycles) considered ideal for intrauterine insemination (age ≤35 years, unexplained infertility, ovarian factor infertility or minimal endometriosis, and a partner with sperm count ≥2.5×10^6^ retrieved on the day of insemination) the pregnancy rate was 12.74%.

**Conclusion:**

In the studied group, female patient age was the only variable significantly correlated with intrauterine insemination success rates.

## INTRODUCTION

Infertility is a public health concern that drives many couples to seek assisted reproductive technology (ART) therapies. However, due to the high cost of *in vitro* fertilization (IVF), less invasive and more affordable procedures such as intrauterine insemination (IUI) have become more popular (Ashrafi *et al.*, 2013; Fauque *et al.*, 2014). However, the distress associated with the treatment should be considered, since the procedure involves a strong emotional investment for the couple. Therefore, it is important to find the prognostic factors favoring IUI over IVF.

IUI is indicated in cases of unexplained infertility, male subfertility, unilateral tubal blockage, cervical or ovulatory dysfunction, and mild or minimal endometriosis (Azantee *et al.*, 2011; Fauque *et al.*, 2014). Despite the improvements in semen preparation and controlled ovarian stimulation techniques, the success rates reported for IUI are lower than the rates reported for other ART procedures (Ghaffari *et al.*, 2015). Data from the European Society of Human Reproduction and Embryology showed that the pregnancy rate per cycle has remained stable for years at 12.4% (Ferraretti *et al.*, 2012; Dinelli *et al.*, 2014). Global pregnancy rates as high as 30% have been reported in some studies on IUI, although results vary depending on the population studied (Luco *et al.*, 2014; European IVF-Monitoring Consortium, 2016).

Regardless of the treatment used, couples are keen to know their chances of success. Therefore, it is crucial to identify and assess the factors that influence the attainment of pregnancy (Ghaffari *et al.*, 2015). Several prognostic factors linked to the outcome of IUI have been identified and related to type of ovarian stimulation and couple characteristics such as female patient age, type and duration of infertility, number of mature follicles recruited, endometrial thickness, number of sperm with progressive motility, sperm morphology, and number of sperm used in insemination (Dinelli *et al.*, 2014; Fauque *et al.*, 2014; Ghaffari *et al.*, 2015).

Since 1992, the Assisted Reproduction Service of the Hospital das Clínicas of the Ribeirão Preto Medical School of the University of São Paulo (HC-FMRP/USP) has offered ART and IUI with patients paying only for the medication used in the procedures. The service has recorded strong inflows of patients from the countryside of the State of São Paulo and other parts of Brazil. The high demand for the services at hand has fostered the development of systems to track the cost of each procedure. In order to encourage the use of low complexity over high complexity techniques, the clinical pregnancy rates of patients offered IUI from 2011 to 2015 in the Assisted Reproduction Service at HC-FMRP/USP was analyzed. Clinical pregnancy rates were then compared for ovulation induction method, cause of infertility, female patient age, number of mature follicles and sperm with progressive motility, in order to determine possible prognostic factors.

## MATERIAL AND METHODS

### Patients and study design

This retrospective observational study was carried out from an analysis of the data collected from 237 IUI cycles performed in 198 women treated from February of 2011 to December of 2015 in the Assisted Reproduction Service at HC-FMRP/USP. The study was conducted in accordance with the guidelines set by the ethics committee at HC-FMRP/USP and the tenets of the Declaration of Helsinki. The ethics committee approved the study design, and the need to obtain informed consent was waived due to the retrospective nature of the study. The included couples had been diagnosed with infertility and were assessed to determine the cause of infertility. The tests included seminograms to assess semen quality, hormone measurements to evaluate the presence of ovulation or menstrual disorders, analysis of the uterine cavity and tubal patency using pelvic ultrasonography and hysterosalpingography and/or hysteroscopy and videolaparoscopy. Assessment of vaginal infection through cytology and detection of couple viral infections through serology tests were also conducted. Treatments and ART procedures were chosen once the cause of infertility was established. Couples prescribed IUI had to have at least one permeable tube of normal diameter and a motile sperm concentration of 5×10^6^/mL on the day of the seminogram.

### Ovulation induction and insemination

The induction of ovulation for IUI was performed according to the standard protocols in effect at the hospital: protocol 1 consisted of Clomiphene Citrate at a dose of 50 to 100 mg/day for 5 days from the second or third day of the menstrual cycle, alone or combined with Gonadotropins - Follicle Stimulating Hormone (FSH) and Luteinizing Hormone (LH) - (Menopur^®^) at a dose of 75 IU on alternate days from the second day of ovulation induction; protocol 2 consisted of Gonadotropins (Menopur^®^) at a dose of 75 IU or recombinant FSH (Gonal^®^ or Puregon^®^) at a dose of 50 to 75 IU on consecutive or alternate days from the second or third day of the menstrual cycle. Ovulation was monitored with transvaginal pelvic ultrasound, starting on the eighth day from the start of ovulation induction medication (Protocols 1 or 2). When a follicle reached a mean diameter of 17 to 18 mm, Human Chorionic Gonadotropin (hCG) (Choriamon^®^) was administered at a dose of 5000 IU or recombinant hCG (Ovidrel^®^) was administered at a dose of 250 mg for oocyte maturation, followed by IUI after 36 to 40 hours. For cases of ovulation induction for IVF using Gonadotropins at 150 to 300 IU/day, IUI was offered only when one or two follicles were recruited or when the patient had patent fallopian tubes and her partner had a motile sperm count of 5×10^6^/mL on the day of the seminogram. The luteal phase was supplemented with Utrogestan^®^ 200 mg or Duphaston^®^ 20 mg per day. Only 12 cycles occurred with no supplementation in the luteal phase.

### Semen preparation

Semen preparation for IUI was performed through sperm washing or density gradient centrifugation. The first method was used for samples with sperm concentration <10×10^6^ regardless of motility, for samples with ≥50% immotile sperm regardless of concentration, and for thawed semen. Semen was added to Human Tubal Fluid (HTF) - 4-(2-hydroxyethyl)-1-piperazineethanesulfonic acid (HEPES) (Irvine Scientific) supplemented with 10% Serum Substitute Supplement (SSS) (Irvine Scientific) at the same proportion; the samples were then homogenized. The samples were centrifuged for 10 minutes at 1000 rpm. The supernatant was discarded and the resulting pellet diluted in 0.5 mL HTF-HEPES + 10% SSS.

The second method consisted of two protocols: 1.0 mL of colloidal suspension was used for samples with progressive motility >32% and 0.5 mL for samples with progressive motility <32%. According to the protocol, 1.0 mL or 0.5 mL of 90% colloidal gradient was first added to the tube and 1.0 mL or 0.5 mL of 45% colloidal gradient was pipetted carefully onto the wall of the tube. Afterwards, a maximum of 3.0 mL of liquefied semen was deposited gently on top of the solution. The sample was centrifuged for 30 minutes at 1000 rpm, the supernatant discarded, and the pellet homogenized in 2.0 mL of HTF-HEPES + 10% SSS medium. A second centrifugation was performed to eliminate residual particles from the colloidal gradient, the supernatant was discarded, and the resulting sediment diluted in 0.5 mL of HTF-HEPES + SSS 10%.

After sperm preparation, the new concentrations and sample motility were determined. The number of sperm with progressive motility to be inseminated was then calculated. Using a 1.0 mL syringe, a LABORATOIRE CCD (Paris-France) insemination catheter was filled with the resulting semen sample. The procedure was performed with the aid of abdominal and pelvic ultrasound guidance.

### Statistics

Software package SAS version 9.3 (SAS Inc Cary, CN) was used for data analysis, with the level of significance set at *p*<0.05. Exploratory data analysis was performed using measurements of central tendency and scatter. Qualitative variables were described in terms of absolute numbers and proportions. Student's t-test was used to compare the groups with regard to quantitative variables. The distribution of variables was assessed through normal probability plots. The chi-square test was used for qualitative variables to test the null hypothesis of absence of association between qualitative variables and clinical pregnancy rates.

## RESULTS

Two hundred and thirty-seven IUI cycles were performed, and 33 patients (14%) underwent more than one cycle (two to four cycles). Clinical data such as patient age, number of follicles recruited on the day of hCG, and semen characteristics on the day of insemination are presented in Table 1.

**Table 1:** Clinical and laboratory characteristics of 237 Intrauterine Insemination cycles

**Variable**	Mean(±SD*)	**Median**	**Q1†**	**Q3‡**	**Minimum**	**Maximum**
Age (years)	36.32 (5.1)	36	33	40	23	48
Concentration sptz pre processing (x10^6^)	81.09 (59.73)	65.5	37.5	117	1.8	313.5
Progressive motility	28.4 (14.01)	28	19	38	0	66
Concentration sptz post processing (x10^6^)	74.86 (58.27)	62	32.5	103	0.2	329.5
Progressive motility post processing (x10^6^)	58.84 (14.88)	60	51	70	8	91
Concentration of recovered sptz	23.83 (21.93)	18.2	7.5	31.98	0.08	131.8
Number of mature follicles (≥17 mm)	1.14 (0.38)	1	1	1	1	3

Sptz=sperm,

* Standard deviation,

† First quartile,

‡ Third quartile

The overall clinical pregnancy rate was 7.59% (n=18 cycles). Laboratory and clinical parameters of the IUI cycles with regard to pregnancy outcomes are presented in Table 2. With the exception of age, there was no significant difference between the groups.

**Table 2:** Clinical and laboratory characteristics of 237 Intrauterine Insemination cycles versus pregnancy outcome

	**Pregnancy**		
	**No**	**Yes**	***P*-value**
Age	36.63 (5.03)	32.56 (4.64)	0.001
Concentration sptz preprocessing	79.4 (57.07)	101.64 (85.25)	0.129
Progressive motility pre processing	28.26 (14.15)	30 (12.39)	0.614
Concentration sptz post processing	74.82 (58.55)	75.38 (56.4)	0.971
Progressive motility post processing	58.58 (15.06)	62.19 (12.12)	0.351
Concentration of recovered sptz	23.75 (22.02)	24.88 (21.39)	0.841

Sptz=sperm

Quantitative analysis of the mature follicles recruited (≥17 mm) during ovulation induction was conducted and results were compared for clinical pregnancy outcomes. Two or more mature follicles were recruited in only 30 patients (12.6%). Among the patients who became pregnant, 16 had one mature follicle and two had two mature follicles.

In 75.5% of the cases (n=179 cycles), ovulation induction was indicated and performed for IUI. IVF was initially indicated in 24.5% of cases (n=58 cycles), but IUI was performed due to low follicular recruitment. The data on the cycles of these two subgroups are shown in Table 3.

**Table 3:** Clinical and laboratory characteristics of 237 intrauterine insemination cycles versus type of ovulation induction

	**Ovulation monitoring for IUI (N=179)**	**Ovulation monitoring for IVF (N=58)**	***P*-value**
Number of mature follicles (≥ 17 mm)			0.5789
1	155 (86.59)	52 (89.66)	
2	21 (11.73)	6 (10.34)	
3	3 (1.68)	0 (0.00)	
Age	35.41 (5.16)	39.12 (3.73)	0.0001
Sptz progressive motility	59.11 (14.71)	58.03 (15.45)	0.8437
Concentration of recovered sptz	24.27 (21.89)	22.49 (22.21)	0.7493

Sptz=sperm, IUI=intrauterine insemination, IVF=*in vitro* fertilization

The causes of infertility were categorized into seven subgroups. Table 4 shows that none of the causes was statistically correlated with clinical pregnancy rates.

**Table 4:** Causes of infertility of 237 cycles of intrauterine insemination versus pregnancy outcome

**Causes of infertility**	**IUI cycles (N=237)**	**Clinical pregnancy (N=18)**
**Unexplained infertility**	60 (25.32%)	5 (27.78%)
**Low ovarian reserve**	45 (18.99%)	2 (11.11%)
**Ovulation factor**	41 (17.3%)	6 (33.33%)
**Male factor**	39 (16.46%)	2 (11.11%)
**Endometriosis**	31 (13.08%)	1 (5.56%)
**Others**	16 (6.75%)	2 (11.11%)
**Tubal factor**	5 (2.11%)	0

IUI = intrauterine insemination

**Figure 1:**
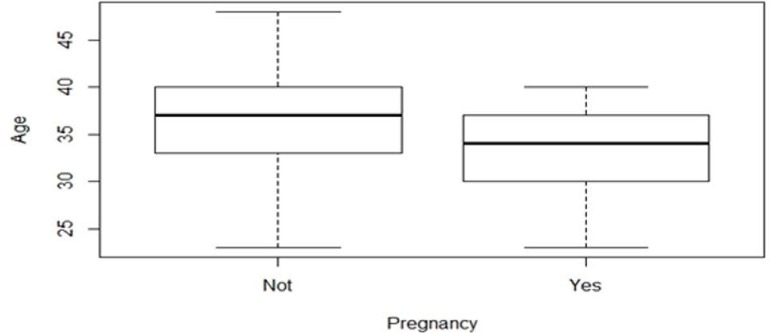
Mean age of patients submitted to intrauterine insemination versus pregnancy outcome

Age was the only variable to present a statistically significant correlation (*p*=0.001) with pregnancy. The mean age of the patients who achieved pregnancy was 32.56 (±4.64) (Figure 1). Clinical pregnancy rates from IUI had no statistically significant association with cause of infertility, number of mature follicles or sperm with progressive motility.

The subgroup of patients showing ideal conditions to undergo IUI was described as having age ≤ 35 years and causes of infertility including unexplained infertility, ovarian factor infertility, minimal endometriosis, and partners with recovered sperm counts ≥2.5×10^6^ on the day of insemination. In this subgroup, 102 IUI cycles were performed, or 43% of all cycles, resulting in a pregnancy rate of 12.74% (n=13 cycles).

## DISCUSSION

Various parameters were analyzed in this study, including female patient age, ovulation induction method, cause of infertility, number of mature follicles, and number of sperm with progressive motility, but only age was significantly related to successful IUI.

Although ovulation was induced based on the procedure indicated for each patient (IUI or IVF), no difference was found in the outcome of pregnancy between induction methods. Fifteen clinical pregnancies occurred in the 179 cycles (83.33%) indicated for IUI, whereas three pregnancies occurred in the 58 cycles indicated for IVF (16.67%). This finding agrees with two studies that showed no significant differences in chemical and clinical pregnancy rates between intracytoplasmic sperm injection and patients converted to IUI (Shahine *et al.*, 2009; Shohieb *et al.*, 2012). Another study reported higher clinical pregnancy rates among individuals converted from IVF to IUI than in the group offered IVF. This suggests that conversion from IVF to IUI is a valid alternative for poor respondents (Freour *et al.*, 2010). In contrast with these findings, a 2010 study showed that, in certain cases, IVF yielded higher pregnancy rates when compared to conversion to IUI (Norian *et al.*, 2010). As illustrated by these studies, there is still no consensus in the literature regarding conversion to IUI for cycles with induced ovulation and poor response during follicular recruitment in ovulation induction for IVF.

Patient age was the only statistically significant variable observed in our study. In line with our findings, previous studies have shown that there is a decrease in clinical pregnancy rates as the age of patients undergoing IUI increases (Zadehmodarres *et al.*, 2009; Ghaffari *et al.*, 2015; Honda *et al.*, 2015). Merviel *et al.* (2010) and Azantee *et al.* (2011) reported that patients aged <30 years have a better chance of achieving pregnancy. In our study, the patients considered ideal for IUI were aged ≤35 years and yielded a pregnancy rate of 12.74%. Ashrafi *et al.* (2013), however, found no association between declining pregnancy rates and increasing age for women aged <40 years, indicating that IUI is also a good alternative for patients aged 40 years and younger. Moreover, some studies have emphasized that female patient age is the most important prognostic factor in the success of IUI after a period of infertility (Yousefi & Azargon, 2011). However, some studies found no association between patient age and outcomes of clinical pregnancy for IUI (Ibérico *et al.*, 2004; Erdem *et al.*, 2008; Akl *et al.*, 2011; Wu *et al.*, 2013).

Causes of infertility were not related to successful pregnancy in our study group. Some studies have found higher rates of clinical pregnancy with IUI in patients with unexplained infertility (Merviel *et al.*, 2010; Azantee *et al.*, 2011; Ombelet, 2013), moderate masculinity, and ovulatory disorders (Merviel *et al.*, 2010; Azantee *et al.*, 2011; Dinelli *et al.*, 2014; Ghaffari *et al.*, 2015). Causes of infertility such as endometriosis and tubal factor, however, produced negative effects and lower pregnancy rates after IUI (Merviel *et al.*, 2010; Wu *et al.*, 2013; Ghaffari *et al.*, 2015).

The number of recruited mature follicles was not correlated with clinical pregnancy rates in this study, although the proportion of cycles with more than one recruited follicle was very low, possibly due to the low doses of ovulation induction drugs used in our protocols. Interestingly, among the cycles that achieved clinical pregnancy, approximately 89% had only one mature follicle. In agreement with our study, Akl *et al.* (2011) and Wu *et al.* (2013) did not find significant differences between the results of IUI and the number of mature follicles. However, these studies revealed that greater numbers of mature follicles were correlated with higher clinical pregnancy rates. A study conducted in 2013 reported a pregnancy rate of 22.5% in cycles with three preovulatory follicles and of 6.5% in cycles with a single follicle (Ashrafi *et al.*, 2013). In a cross-sectional analysis, Ibérico *et al.* (2004) reported that the application of IUI in cycles with three mature follicles almost tripled pregnancy rates when compared to cycles with only one follicle. In a literature review, Merviel *et al.* (2010) reported that the strongest predictive factor for pregnancy after IUI was ovulation stimulation enabling the recruitment of at least two follicles >16 mm.

Our study also looked into the pre- and post-processing number of sperm with progressive motility, and found no association between these parameters and clinical pregnancy rates. Accordingly, studies by Akl *et al.* (2011), Ghaffari *et al.* (2015), Luco *et al.* (2014), and Ombelet *et al.* (2014) indicated that sperm parameters did not significantly affect IUI success. In contrast, other authors including Azantee *et al.* (2011) reported that sperm parameters were correlated with IUI success, adding that one of the more significant prognostic factors for the success of the procedure was semen quality, as patients with low sperm counts (oligospermia) and low counts of sperm with progressive motility (asthenozoospermia) had more adverse IUI results (Barros Delgadillo *et al.*, 2006).

Another variable that might influence the outcome is total motile sperm count (TMSC), which is the product of the sperm volume collected based on sperm concentration and the percentage of sperm with progressive motility in the ejaculate (Nikbakht & Saharkhiz, 2011). However, due to a partial lack of data, this variable was not calculated in our study. Some authors reported that higher total motile sperm counts lead to greater likelihood of pregnancy after IUI (Ibérico *et al.*, 2004; Merviel *et al.*, 2010; Yousefi & Azargon, 2011). Nikbakht & Saharkhiz (2011) evaluated the prognostic value of TMSC and the number of motile sperm inseminated (NMSI) in IUI, and showed that pregnancy rates were higher when the TMSC ranged between 5×10^6^ and 10×10^6^ (15%), and lower in subgroups that had counts <1×10^6^, from 1×10^6^ to <5×10^6^, and ≥10×10^6^ (5.6%; 5.1%; 10.8% respectively). NMSI ≥10×10^6^ resulted in higher pregnancy rates (11.2%) versus subgroups with counts <5×10^6^ and from 5×10^6^ to <10×10^6^ (4.1% and 5.2%, respectively). Supporting Nikbakht & Saharkhiz (2011), Lemmens *et al.* (2016) reported that IUI is particularly relevant for couples with NMSI ranging from 5 to 10×10^6^.

It has been shown that pregnancy rates may vary significantly depending on the number of motile sperm inseminated (Badawy *et al.*, 2009; Merviel *et al.*, 2010; Cao *et al.*, 2014). Dinelli *et al.* (2014) reported that the main problem related to male infertility was moderated asthenozoospermia, and that pregnancy rates were significantly higher when the number of motile sperm with progressive motility used in insemination was at least 1×10^6^.

Finally, although the clinical pregnancy rate seen in our population was 7.59%, the subgroup of patients thought to have ideal conditions for intrauterine insemination (age ≤ 35 years, unexplained infertility, ovarian factor infertility or minimal endometriosis, and partners with sperm count ≥2.5×10^6^ retrieved on the day of insemination) had a pregnancy rate of 12.74%. Geisler *et al.* (2017) recently aimed to find the factors that might support more individualized applications of IUI, and reported that IUI associated with controlled ovarian hyperstimulation, especially in younger patients, produced good live birth rates. More than 90% of the live births with IUI were achieved during the first two cycles. Our findings for this age group were similar, in that younger patients had better pregnancy results with IUI. These findings suggest that probabilities of success may be used to individualize treatment decisions, while IUI before IVF for carefully chosen patients still is a treatment option.

A possible limitation of our study was the size of the sample, which may not have been large enough to detect differences in some of the analyzed parameters. Further studies with larger sample sizes may be necessary to confirm the results of this study.

## CONCLUSION

The clinical pregnancy rate found in this study was 7.59%. When ideal conditions were present for the indication of IUI, the pregnancy rate was 12.74%. Female patient age was the only variable significantly associated with IUI success. Ovulation induction method, cause of infertility, number of mature follicles, and number of sperm with progressive motility were not associated with pregnancy outcome. Due to affordability and when accompanied by appropriate patient selection, IUI remains an effective method among the available options for infertile couples.
